# Pregnancy in HIV Clinical Trials in Sub Saharan Africa: Failure of Consent or Contraception?

**DOI:** 10.1371/journal.pone.0073556

**Published:** 2013-09-10

**Authors:** Agnes Ssali, Stella Namukwaya, Leonard Bufumbo, Janet Seeley, David G. Lalloo, Anatoli Kamali, Rosalind Parkes-Ratanshi

**Affiliations:** 1 MRC/UVRI Uganda Research Unit on AIDS, Uganda Virus Research Institute (UVRI), Entebbe, Uganda; 2 Family Health International 360, Kampala, Uganda; 3 Liverpool School of Tropical Medicine, Liverpool, United Kingdom; 4 Infectious Diseases Institute, Kampala, Uganda; 5 School of International Development, University of East Anglia, Norfolk, United Kingdom; Fundacion Huesped, Argentina

## Abstract

**Objective:**

Higher than expected pregnancy rates have been observed in HIV related clinical trials in Sub-Saharan Africa. We designed a qualitative study to explore the factors contributing to high pregnancy rates among participants in two HIV clinical trials in Sub-Saharan Africa.

**Methods:**

Female and male participants enrolled in one of two clinical HIV trials in south-west Uganda were approached. The trials were a phase III microbicide efficacy trial among HIV negative women using vaginal gel (MDP); and a trial of primary prevention prophylaxis for invasive cryptococcal disease using fluconazole among HIV infected men and women in Uganda (CRYPTOPRO). 14 focus group discussions and 8 in-depth interviews were conducted with HIV positive and negative women and their male partners over a six month period. Areas explored were their experiences about why and when one should get pregnant, factors affecting use of contraceptives, HIV status disclosure and trial product use.

**Results:**

All respondents acknowledged being advised of the importance of avoiding pregnancy during the trial. Factors reported to contribute to pregnancy included; trust that the investigational product (oral capsules/vaginal gel) would not harm the baby, need for children, side effects that led to inconsistent contraceptive use, low acceptance of condom use among male partners. Attitudes towards getting pregnant are fluid within couples over time and the trials often last for more than a year. Researchers need to account for high pregnancy rates in their sample size calculations, and consider lesser used female initiated contraceptive options e.g. diaphragm or female condoms. In long clinical trials where there is a high fetal or maternal risk due to investigational product, researchers and ethics committees should consider a review of participants contraceptive needs/pregnancy desire review after a fixed period, as need for children, partners and health status of participants may alter over time.

## Introduction

Sub Saharan Africa has the highest fertility rate of any world region, at 5.77 children born per woman [1]. In Uganda the fertility rate in 2009 was 6.7 children born per woman [2]. Whilst contraceptive use is increasing by 0.7% per year across the continent, many women have difficulties in accessing contraceptives; it is estimated that 41% of women in Uganda have an unmet need for contraception [3].

In couples affected by HIV desire for pregnancy and contraceptive needs are complex issues [4]. A Ugandan study of desire for children and pregnancy risk in HIV infected men and women showed that 27% of men reported wanting more children. Of the 73% of men who reported that they did not want more children 33% still practiced “risky” sexual behaviour (defined as sex without using condoms) [5]. Another study in Uganda showed that 59% HIV positive women in HIV sero-discordant couples desired to get pregnant [6]. Among women on antiretroviral therapy in rural Uganda followed for 2.4 years, only 7% of 711 women reported wanting more children at any time point, however by the end of the follow up period 16.9% women had become pregnant [7]. In Rwanda and Zambia, contraceptive knowledge, use and concerns amongst HIV discordant couples were examined and despite high levels of knowledge of contraception 90%, use of contraceptive methods remains relatively low (at between 30–59%) [8].

In clinical research, pregnancy is an exclusion criterion for participation in most trials [9], [10]. Often becoming pregnant once enrolled in a trial is classified as an adverse event and leads to withdrawal of participation from the trial. A higher than expected pregnancy rate within a trial may have an impact on the analysis as the withdrawal of participants may affect the statistical power of a study. Therefore, there has been a lot of interest in observations that HIV clinical trials involving women of reproductive age in Sub Saharan Africa have high pregnancy rates. In many HIV microbicide gel studies, for example, the researchers undertake continuous education and promotion of consistent use of condoms for HIV prevention, and condoms for contraception are often supplemented with the addition of a second hormonal contraception. Despite these measures pregnancy rates of 21–76 pregnancies per 100 person years have been reported in these trials [11]–[13].

These observations have led to work on assessment of contraceptive use in microbicide and vaccine study participants but there is little depth analysis on the socio-cultural factors determining pregnancy in women during HIV clinical trials in Africa.

Between 2003 and 2007 two HIV related clinical trials took place in Masaka district in South Western Uganda. One study was the primary prophylaxis of cryptococcal disease using fluconazole in HIV infected Ugandan adults (CRYPTOPRO – ISRCTN76481259). The second trial was the Masaka site of Microbicide Development Programme trial, an international multi-center phase III to evaluate the efficacy and safety of 0.5% and 2% PRO 2000/5 gel compared to a placebo for the prevention of vaginally acquired HIV infection (MDP-ISRCTN 64716212). The methodologies of these have been previously published [14], [15]; in summary the CRYPTOPRO trial included both HIV positive women and men (above 18 years of age), whereas the MDP trial enrolled sero-discordant couples, comprised of HIV negative women (above 18 years of age) and HIV positive men. In CRYPTOPRO participants were followed up for a period of up to 24 months and 5.4 % (N = 54) of 982 HIV positive women became pregnant. There were 21 live births and there was no evidence of fluconazole related abnormalities in live born babies [14]. In MDP the participants were followed up for a maximum period of 24 months and the pregnancy rate in this study was 16.3% (N = 137) of the 840 HIV negative women. In SSA HIV positive women with low CD4 counts have reduced fertility, as compared to HIV negative women [16] therefore the difference in pregnancy rates between these group is not surprising, but both trials experienced a higher than expected pregnancy rate.

In this paper we present a study designed to investigate issues surrounding attitudes, knowledge and feelings about the main contributing factors leading to pregnancy or avoidance of pregnancy of participants in these two trials. This study was designed to evaluate views of both HIV positive and negative women enrolled in the trials and also their male partners.

## Methods

### CRYPTOPRO and MDP Trial Consent Process with respect to pregnancy and contraception

Prior to enrolment in CRYPTOPRO, female and male participants were counselled by trained counsellors about the possible but unlikely risk of fluconazole causing congenital abnormalities. In the MDP trial couples were enrolled together and both were counselled that the effects of gel on pregnancy are not known and gel did not act as a contraceptive method. In both trials at enrolment and at every appointment (every 8 weeks for CRYPTOPRO and every 4 weeks for MDP) the women underwent a pregnancy test, were counselled about avoiding pregnancy and were offered various contraceptive methods. Those who had a positive pregnancy test on either study were withdrawn from trial products. Pregnant women were given an appointment by an independent physician during pregnancy and infants were seen by a paediatrician. Follow up continued until after the end of their pregnancy.

### Recruitment for the pregnancy study

**Figure 1:**
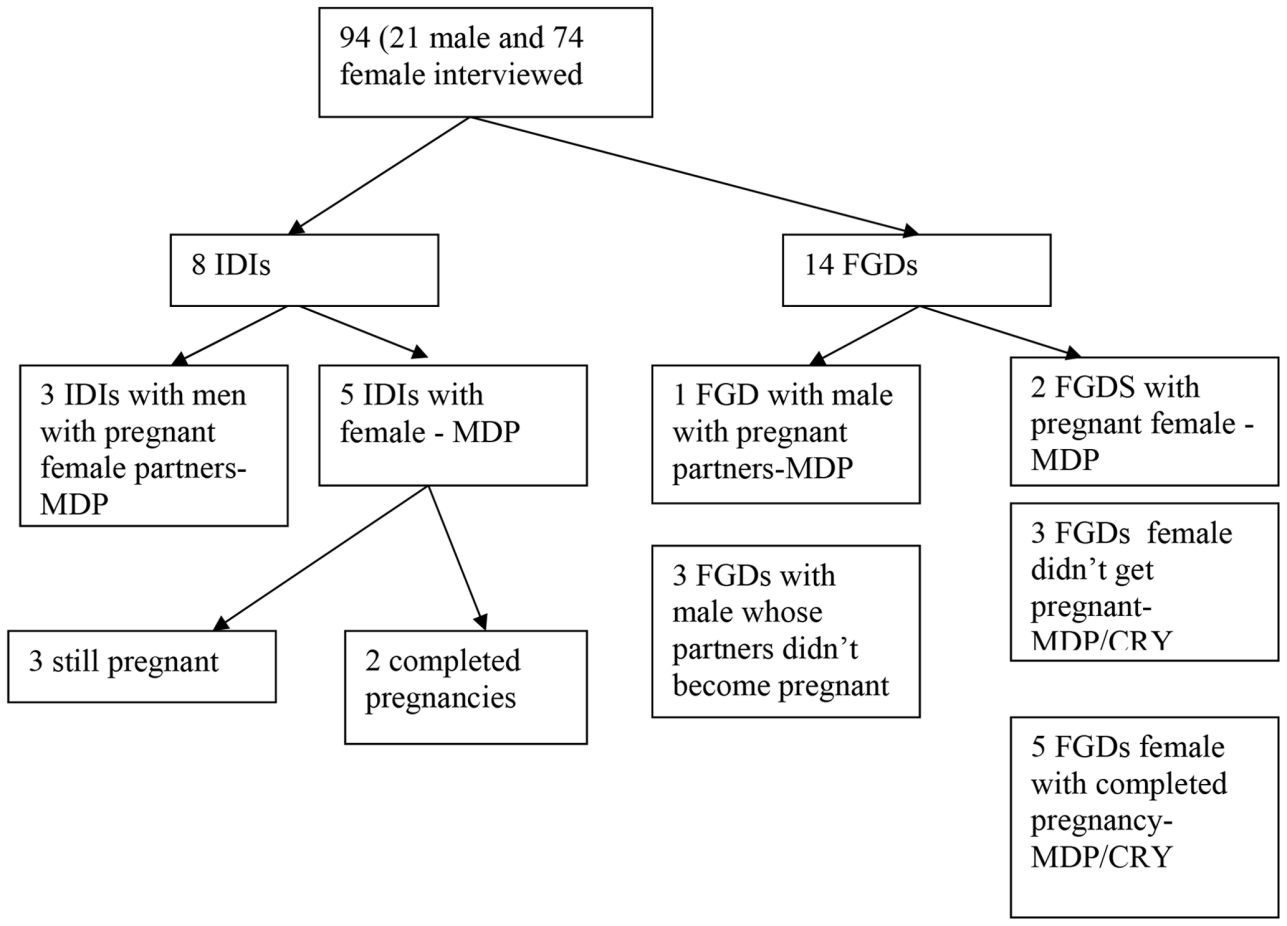
The distribution of participants in the study.

HIV positive female participants in the CRYPTOPRO trial and HIV negative female participants in the MDP trial were invited to join this study. Female participants who were currently pregnant at the time of the pregnancy study or who had completed a pregnancy during the trials were identified through the CRYPTOPRO and MDP databases. These trials recruited from the whole Masaka region, which is a large rural region of South West Uganda, with poor infrastructure, and so we chose purposive sampling. We then identified non-pregnant female MDP and CRYPTOPRO participants living within similar vicinity as the pregnant women, as additional potential enrolees to the study. The female participants were approached at their homes (consent for this had been given at trial enrolment) and given brief information about the pregnancy study. They were then asked to attend a specific appointment where they were given more detailed information about the study, their questions were answered by the study team and those who agreed to participate gave written informed consent for inclusion in the pregnancy study. Of 130 MDP and CRYPTOPRO trial participants approached, 94 were enrolled in the sub study. Those who did not take part had either moved, were not available at time of study or were unable to make it for the FGDs. Two female participants refused consent. The distribution of participants and focus groups are shown in figure 1.

In order to recruit men for the study women who enrolled in the pregnancy study were asked if we could approach their male partners for inclusion for this study. 21 male partners agreed to take part in the study; these were HIV positive partners of women in the MDP study and either HIV positive or HIV negative partners of those in the CRYPTOPRO trial. Those who agreed to participate after an information session about the study completed the informed consent process.

### Data collection

Women and male volunteers were invited to participate in focus group discussions (FGDs N = 14; 23 male and 53 female participants) and in-depth interviews (IDIs N = 8; 5 female, 2 of whom were still pregnant and 3 had completed pregnancy and 3 HIV positive male partners from MDP with pregnant partners). The focus groups had between 5–10 participants and on average there were six participants in each FGD. The FGDs were conducted in communities where participants agreed to meet at specified venues. IDIs were conducted at the volunteer's home in privacy. Data collection for this study took 6months (September 2008 to February 2009) to complete. The FGD and IDIs were conducted in the local language of Luganda, by trained interviewers using semi-structured guides. The interviewers were trained and had experience counselling in HIV, contraception and training and experience in qualitative research methods. They had no prior relationship to the participants. The main topics discussed during the FGDs and IDIs were; perceptions about pregnancy, reasons for becoming pregnant, contraceptive use, partner involvement, and issues of consenting to the trials and beliefs about the trial products (gel and fluconazole). The sample size was not predetermined but data was collected with subsequent new participants until there was no new information emerging (but repeat interviews with individuals were not performed).

All collected data was audio recorded and later transcribed into English and coded under the main themes and topics of the study. Comparison of responses from the women who got pregnant and those who never got pregnant were made. Comparison of the responses from male whose partners got pregnant and those whose partners didn't get pregnant were also made. The different responses from the different groups were categorised and analysed by 2 social scientists (AS, SN) and a 3rd researcher (RPR) with the support of the qualitative data analysis software Nvivo 2.

### Ethics Statement

Participants gave written or, if illiterate, witnessed verbal consent (documented on the consent form) to involvement in the study.The study and consent process was approved by the institutional regulatory body of Uganda Virus Research Institute ref; GC/127/07/08/05 and by the Uganda National Council of Science and Technology ref: SS2134.

## Results

The results of this sub-study are shared according to the main themes that came out of the discussions and in-depth interviews with the participants. We have attempted to analyse the issues according to HIV status and gender issues, including complexities around discordant couples.

### Did the participants understand the consent process?

All MDP and CRYPTOPRO participants who were interviewed in this study acknowledged the importance of avoiding pregnancy during the trial. Most women mentioned that they had been given pregnancy counselling and mentioned information sheets given to them to explain that the products in the trials had never been tested on pregnant women and that becoming pregnant during the trial would lead to discontinuation of participation. The women interviewed who became pregnant during the MDP trial reported that they did not think the gel would affect their unborn babies and this was reinforced by normal pregnancy outcomes in themselves and in their colleagues:

*“I think that the drug was okay because for me I used it while pregnant and it did not affect me. I took a full month taking it because I was not convinced that I was pregnant.* –Female HIV+ve with completed pregnancy (CRYPTOPRO).

*“At this moment we have no proof of whether the drug deforms a baby but the woman was in the trial and she gave birth to a full child without any deformation”* Female HIV +ve not pregnant (CRYPTOPRO).

Whilst all participants interviewed understood the consent process and the majority of women stated that they had no intention of getting pregnant while on the trial, one couple in the MDP trial admitted that although they consented to be in the trial they were still planning to get pregnant:

*“We had a plan; her pregnancy was not by mistake”* HIV+ve male with female HIV –ve pregnant partner (MDP).

### What social and cultural factors surrounding fertility contributed to the couple's use of contraception and fertility outcomes?

Trial participants and their partners from both genders and sero-status (HIV positive and HIV negative men and women) expressed strong desires to have children (if childless), a desire for a certain number of children and for children of a particular sex. These desires overruled the advice given by the study teams about the risk of pregnancy. Additionally many expressed that fertility desires also outweighed the risk of HIV transmission/acquisition:

*“I would want my partner to give birth to some more children for me to add on because now I have one boy and girl so I would want my partner … to have another boy”* Male partner of female HIV+ve not pregnant (CRYPTOPRO).

*“The man had only two children and we had total disagreement between us about using condoms, even after l was found HIV positive, so I eventually got pregnant”* Female HIV +ve with completed pregnancy (MDP).

Beliefs of other family members and wider cultural beliefs also played a role in the desire to have another child:

*“When l told my mother that l was using family planning methods, she advised me to have another child because my last pregnancy l bore twins. She said a woman must have another child after the twins, otherwise the twins will be cursed and get problems”.* Female HIV-ve completed pregnancy (MDP).

In the MDP trial HIV negative women were enrolled with their HIV positive male partners and the men were aware of gel use by the women. Anxieties were expressed by HIV positive men in this trial about the fear of relationship breakdown due to the pressure of a being a discordant couple. Becoming pregnant was seen as a mitigating factor to relationship breakdown in some couples:

*“Another thing is that you may have just got your partner and you may have got her with difficulty, and you don*'*t have a child, so… you want her to give birth”* HIV+ve male with female HIV –ve pregnant partner (MDP).

In contrast to the MDP trial all participants in the CRYPTOPRO trial were HIV positive, and often in very poor health with low CD4 counts at trial enrolment. Those who started on antiretroviral therapy (ART) consequently had an improvement in their health. This is likely to have increased their fertility [16], and also these improvements in health encouraged both female participants and their male partners to consider pregnancy whilst still within the trials:

*“When these men see that you have been taking ARV*'*s and are looking healthy, they start telling you to have babies”* Female HIV+ve completed pregnancy (CRYPTOPRO).

However, some participants expressed that other community members have negative views about pregnancy in those who are known to be HIV positive:

*“When I got pregnant people were surprised and it is embarrassing for us because they see the man as a dead man”* Female HIV-ve completed pregnancy (MDP).

HIV positive women in the CRYPTOPRO trial explained that they did not access contraception if they were not with a regular partner, so may have unprotected sex with a casual or infrequent partner. Stigma surrounding HIV status contributed to lack of disclosure; some explained that they could not discuss contraception with their partner, as they had not disclosed their serostatus:

*“He does not know about my HIV serostatus and l do everything secretly because we do not live together all the time”* Female HIV+ve completed pregnancy (CRYPTOPRO).

### How did women's experiences and beliefs surrounding contraceptive methods affect adherence to contraception?

#### Negative views and experiences

In both trials the main methods of contraception reported by the women were the progesterone injection, hormonal contraceptive pills, intrauterine contraceptive devices (IUCD) and condoms. Most women reported that they got their contraceptive supplies from the trial teams, or from other health care facilities. All of the women interviewed acknowledged that they understood the need for contraceptives.

Many women in the trials suffered from well documented and frequently reported side effects of hormonal contraception. These included irregular or longer periods, ammenorrhoea and weight gain. Some women persevered and another method hormonal contraception, which suited them better:

*“I used to use injectable (Depo-Provera) but l got so fat we started using condoms but one time it got torn”* Female HIV-ve completed pregnancy (MDP).

Use and knowledge of traditional methods or “herbs' was frequently mentioned, either as an alternative to hormonal contraception, with less side effects, or as an adjunct to condoms alone. Use and knowledge of these medicines was from other female community members:

*“I got the injection from the clinic…but I got side effects due to that injection. That*'*s why they directed me to get some local herbs….but I don*'*t how it failed and I got pregnant again”* Female HIV +ve pregnant (CRYPTOPRO).

Women reported that men were unwilling to use condoms, or inconsistently used them. The reasons for this were multifactorial and included alcohol use, desire for more children, or lack of enjoyment. Some women believed that men had even purposefully damaged a condom in order for their partners to become pregnant:

*“He either did not put on the condom which I didn*'*t realize or he pierced it”* Female HIV-ve with completed pregnancy (MDP).

#### Positive views and experiences

Whilst a number of women in the trials had poor experiences with contraception, there were many women who had positive experiences with family planning, and who were prepared to make considerable efforts to avoid pregnancy. Some women reported saving money for transport to the clinic to refill/renew their contraceptives. They felt that they had to take this responsibility themselves, and relying on partners to give them money was not sufficient. Women also acknowledged that dual methods (condom plus one other) of contraception were both more effective than a single method, and gave greater reassurance if a woman was anxious to avoid pregnancy:

*“At first I used pills but failed but now I use two methods; injections and condoms and the reason why I use two is because sometimes I may fail to get a condom and I put myself at risk but when the injection is there then am not worried”* Female HIV –ve not pregnant(MDP).

Some women who were keen to avoid pregnancy against their partners' wishes used contraception secretly:

*“l did not tell him at first because he did not like the contraceptive pills so l used them secretly but when they started making me sick l had to tell him”* Female HIV –ve completed pregnancy (MDP).

Both positive and negative views of women regarding contraception as outlined above were similar in both the HIV positive CRYPTO participants and HIV negative MDP female participants. However, an additional issues raised by participants in the CRYPTO trial was a correct acknowledgement that hormonal contraception may not be as effective in preventing pregnancy in HIV positive women those taking ART. This led to some not trusting the hormonal contraception, and being unwilling to continue use of these methods.

### The male perspective on family planning in the context of clinical trials

Male partners were aware of various contraceptive methods, but the responsibility of accessing hormonal contraceptives and using them appropriately was deemed to be the responsibility of the women. The men views on contraception were mainly limited to the advantages and, more commonly, disadvantages of condom use, as these seemed to have a most significant impact on their sexual experiences. The men appear to be most frequent decision makers when it comes to condom use:

*“The truth is that with a condom sometimes you don*'*t get enjoyment although we try to use them because there is something that we are preventing but they are difficult since it is like eating tasteless food”* Male with female HIV+ve not pregnant partner (CRYPTOPRO).

When hormonal contraception by men was discussed, it was mainly in relation to avoiding condom use:

*“Another thing is that the injection is better than a condom because she receives the injection for three months…… you may leave them at home and when you come back there you go [can have sex]”* Male HIV+ve with female HIV –ve pregnant partner (MDP).

### Facing the consequences –thoughts on unplanned pregnancy

The women reported that they were not happy when the gel or fluconazole was withdrawn due to pregnancy, as they believed that the absence of the trial products would adversely affect their health. Women from the MDP trial expressed fear of contracting HIV once they stopped using gel. Many women reported that gel use was also a means of encouraging negotiation with their regular sexual partners to use condoms. One woman reported that fellow participants laughed at her for getting pregnant. As outlined in section 1, participants in both studies were not concerned about the effect of investigational products on their pregnancies.

Male partners also expressed regrets about their partner's pregnancies whilst on the trial:

*“l felt bad when they withdrew gel but when my wife gives birth l will use the gel again and l will follow instructions”* Male HIV+ve with female HIV –ve pregnant partner (MDP).

### Views on compulsory directly observed contraception within trials

During the consent process, the research teams in the trials emphasized the need for family planning and the different contraceptives methods available to participants. However, during this study it emerged that participants did not consider contraception compulsory. When asked if the women would participate in a trial if they had to use directly observed contraceptives (such as IUD, Implant or depo injection) as a must the response was mixed:

*“For some of us that* (compulsory contraception) *would have destroyed our homes since we have just married and people would start asking what has caused a delay in getting pregnant, and they would say the man wasted his time, and he could end up getting children elsewhere”* Female HIV –ve completed pregnancy (MDP).

*“For me l would have joined because I already have children”* Female HIV –ve completed pregnancy (MDP).

## Discussion

Recent experience in HIV clinical trials in Sub Saharan Africa has shown that many women get pregnant whilst in a clinical trial. This has raised anxiety in researchers that there is a failure of consent, and that trial participants do not fully understand the importance of avoiding pregnancy. Whilst some of the increased rates may be due to earlier diagnosis of pregnancy of both viable and non-viable pregnancies in clinical trials compared to routine care (due to the use of highly sensitive pregnancy tests and more regular visits), but this alone does not adequately explain the high pregnancy rates [17], [18].

This study aimed to explore issues in women and men in two different HIV related trials run concurrently in the same part of Uganda. One was a prevention trial with both male and female, HIV negative and positive participants. The other study was an HIV intervention study with all HIV positive participants. One trial was using a novel product and one using a widely used licensed drug in a novel manner. By gathering information from participants and their partners in two different trials we were attempting to see what general similarities exist around reasons for pregnancy in different research settings in order to help us plan future HIV trials in Uganda which address the contraceptive needs and fertility desires of participants as much as possible. This was a retrospective study so inherently open to bias. We tried to avoid this by choosing of a wide range of different focus group participants. Given the sensitive nature of contraception and pregnancy issues the choice members for each focus groups was designed to maximize the comfort level of the participants (by separating the women into pregnant, non-pregnant and those who had completed their pregnancy) as it was important to try to make the participants of each group as open with each other as possible. The thoughts and desires of men have not been studied in this context, and including male partners was an attempt to get a full picture of decisions that couples make regarding fertility in clinical trials.

We found that the consent processes seemed to be effective in both trials; the participants acknowledged that they had received counselling and consent about adverse or unknown effects of trial product on a pregnancy. However, there was a general lack of concern about the drug affecting the pregnancy; only one mother was concerned about trial drug after giving birth to a low birth weight baby. This is an area we feel that warrants further exploration; is there a cultural belief in Uganda that in general drugs do not harm the unborn child? In diaphragm and gel study in South Africa there was a strong positive feeling towards investigational products in terms of enhanced sexual pleasure and product protection against HIV”[19]. Therefore, perhaps this is part of a positive belief around investigational products “prevention method optimism” as termed by Van der Straten during work in Zimbabwe with the diaphragm as STI prevention [20]. As a result of this we feel that whilst discussions and questionnaires to assess understanding of a research trial may be adequate for most trials, exploration of alternative methods for informed consent evaluation, such as vignette or narratives, as evaluated for HIV vaccine studies in Sub Saharan Africa [21], could be considered in cases where there is a high risk of foetal abnormality or risk to the mother if she becomes pregnant.

Motivation for enrolling in a clinical trial may be multiple and complex. Studies have shown that there may be a desire to get access to improved health care, access to HIV testing or a desire to help the community. In the MDP study protection against HIV transmission for a discordant couple was strong. Occasionally the motivation is financial. Therefore, this may lead to commitment on the part of the study participant that is difficult to keep [22]–[24]. In our study the couple who planned for pregnancy whilst taking part in the MDP trial is an interesting example of this; further exploration of their motivation for joining the trial may have been insightful.

Homsy and colleagues [7] concluded that women and their partners need to be offered regular and comprehensive family planning services as part of their standard package of care to reduce on unwanted/unplanned pregnancies. A randomized intervention looking at pregnancy and HIV/STD risk reduction suggested partner support for contraception may assist in reducing unwanted pregnancy [25]. However, concerns about health and side effects of contraception were expressed strongly by both HIV positive and negative women in this study, and this is in line with other published data suggesting that around 23% of women in Sub-Saharan Africa are not accessing contraception because they are concerned about the health and side effects of contraception [26]. Readily available contraceptive options in this setting are hormonal contraceptives (oral, injectable and implants methods) and non-hormonal methods (IUD and condoms). There is some evidence that African women desire to see a menstrual period, and may be equivocal about injectable and implant hormonal contraceptive use [27]. However, young women in South Africa report high levels on hormonal contraceptive use (66.6%) [28] and the use of injectable contraception is rapidly increasing across the continent [29], [30] (albeit with high discontinuation rates of 25–36% within the first year, due to side effects or wanting to get pregnant). Participants in an HIV vaccine trial in East Africa showed a greater uptake of injectable contraception (34%) as compared to other hormonal contraception (24.3%) [31]. An HIV prevention trial focusing on diaphragm and lubricant gel use highlighted the advantages of female initiated non hormonal contraception, and whilst not currently commonly used in Africa, this work highlights that the diaphragm is worth exploring as alternative method [19].

Ethically, trials that involve investigational products with any possibility of foetal abnormality undertaken in women of childbearing age require protection of the participants against pregnancy. Condom use is increasing in SSA [32], and some microbicide trials have shown high uptake (99%) [33]. Our work shows a reluctance of some men to use condoms consistently, even when they are in a trial and in a discordant relationship. We also know that they are not 100% effective at preventing pregnancy. In addition there is concern over increased HIV transmission and acquisition with hormonal contraception [12], and current WHO guidance is to advise condoms when using hormonal contraception. Hormonal contraceptive and antiretroviral therapy have complex drug interactions. Therefore, we feel that the ethical obligation on the part of researchers to offer condoms plus an additional contraception (ideally chosen by the woman) is appropriate. Given varying cultural contexts and different fertility desires of women, a range of contraceptive options should be available. However, there also needs to be an adequate explanation of the different contraceptive methods offered, with the efficacy and safety profiles in the context of HIV outlined to potential participants.

Finally, it is clear that men also have a role to play in the deciding if a couple gets pregnant. In some circumstances there is also pressure from other family members. HIV clinical trials often recruit women of childbearing age, and participants are often followed up for long periods of time. Perhaps it is unrealistic to ask couples of child bearing age to avoid pregnancy for long periods either in HIV prevention studies when the women may well be HIV negative, or in HIV treatment trials where the women are accessing ART which improves their health. Researchers need to acknowledge this, and encourage an open discussion with participants about contraception and fertility needs; far better a women withdraws from a study with knowledge of researchers, than discontinues trial product use, as we have seen in microbicide trials in South Africa [34].

Given current efficacy and safety issues with different contraception methods in the context of HIV, consent for clinical trials involving HIV positive participants should carefully address these complex contraception issues in order to make sure that participants understand all of the different issues related to hormonal and non-hormonal contraception in this environment.

## Conclusion

Individuals and couples in these trials made decisions to become pregnant despite acknowledging that they received information about risks during the informed consent process. A couple's desire for children changes over time, and with changes in health, financial and relationship status. Researchers and scientists need to take this into account when planning trials in regions with high fertility rates. The level of risk to a pregnancy of investigational products in trials recruiting women of childbearing age needs to be assessed and discussed in ethics committees; with increased contraception options (inclusion of the diaphragm for example), and perhaps a separate consent form for women which more fully explores contraceptive choices (with efficacy and safety). As researchers we need to be realistic about the limitations of informed consent when it comes to something as fluid and complex as the desire, and capacity of a couple to have children; for very high risk products, perhaps a review of informed consent and pregnancy desires on an annual basis should be considered.
